# Jaundice, Coombs Positive Hemolytic Anaemia, and Liver Failure: An Unusual Trio

**DOI:** 10.7759/cureus.33158

**Published:** 2022-12-31

**Authors:** Sulhera Khan, Wajeeha Batool, Rabya M Munir Ahmad, Zeeshan Ali, Syed Masroor Ahmad

**Affiliations:** 1 Department of Internal Medicine, Jinnah Postgraduate Medical Centre, Karachi, PAK; 2 Department of Internal Medicine, Jinnah Sindh Medical University, Karachi, PAK

**Keywords:** hev vaccine, coomb's positive hemolytic anaemia, wilson's disease, acute hepatitis e infection, acute on chronic liver failure

## Abstract

Hepatitis E virus (HEV) is a single-stranded RNA virus with 20 million cases reported worldwide. Infected individuals may either remain asymptomatic or develop acute or even fulminant hepatitis. HEV has been implicated in causing Acute-on-Chronic Liver Failure (ACLF) among patients with underlying cirrhosis. Among the causes of cirrhosis, Wilson's disease is an identified cause that results in an increased accumulation of copper in the liver, brain, and other organs. It is noted that Coombs negative hemolytic anaemia is also seen in the clinical spectrum of Wilson's disease, however, Coombs positivity has not been documented. We present a case of a young female who had an undiagnosed chronic liver disease (CLD). The patient developed acute decompensation with HEV infection along with Coombs positive hemolytic anaemia. Her autoimmune hepatitis screen was negative, so the patient was worked up for other causes of CLD, which led to a diagnosis of underlying Wilson's disease. The patient was started on penicillamine and zinc acetate. However, during the disease, the patient developed acute decompensation and unfortunately expired before her transplant could take place. Our case documentation is of importance as Coombs positivity in patients with Wilson's disease has not been reported before. Attending physicians should be suspicious of Wilson's disease in a patient with Coombs positive hemolytic anaemia when other causes cannot be identified. It is also important to promptly identify any other cause of CLD to educate patients regarding factors leading to acute decompensation and progression to ACLF.

## Introduction

Hepatitis E virus (HEV) is a food- and water-borne viral illness with a worldwide prevalence of 20 million and overall mortality of 70,000 [1]. There are four genotypes of HEV, with genotypes 1 and 2 being common in developing countries as they spread via failure of basic hygiene practices and faecal contamination of drinking water, and genotypes 3 and 4 are also known to infect pigs and other mammals [2]. HEV has a mortality rate of 0.5% to 4% in immunocompetent patients, increasing to 20% in immunocompromised patients, pregnant women (mostly in the second or third trimester), and those with underlying chronic liver disease (CLD) [3]. It is also observed that the mortality associated with HEV infection is greater in developing countries like Pakistan [2]. Patients with underlying CLD may present with acute decompensation secondary to HEV infection. Among the several causes of CLD, Wilson’s disease is one of the recognized causes. It is an autosomal recessive condition, caused by the homozygous mutations in the *ATP7B *gene, leading to a deficiency of serum ceruloplasmin, causing increased accumulation of copper in the liver and other organs, including the brain, joints, and eyes [4]. The prevalence of Wilson’s disease is 1 in 30,000; however, it may be higher due to a high rate of underdiagnosis [5]. Along with neuropsychiatric and hepatic features, patients may develop haematological complications, including Coombs negative hemolytic anaemia [6]. Here, we report a case of a young woman with undiagnosed underlying Wilson’s disease complicated with acute HEV infection associated with Coombs positive hemolytic anaemia, a feature not commonly seen in Wilson’s disease.

## Case presentation

A 16-years old unmarried female, a resident of Pakistan, presented via the outpatient department with a history of fever and yellow discolouration of sclera and skin for three weeks. The fever was low grade without any chills and rigors subsided with antipyretics. The patient denied any associated pruritus, pale stools, dark-coloured urine, and abdominal pain. She had secondary amenorrhea for the past 12 months. Her parents had a consanguineous marriage. She belonged to a low socioeconomic class with poor sanitation at home. Her drug, transfusion, and travel history were insignificant.

**Figure 1:**
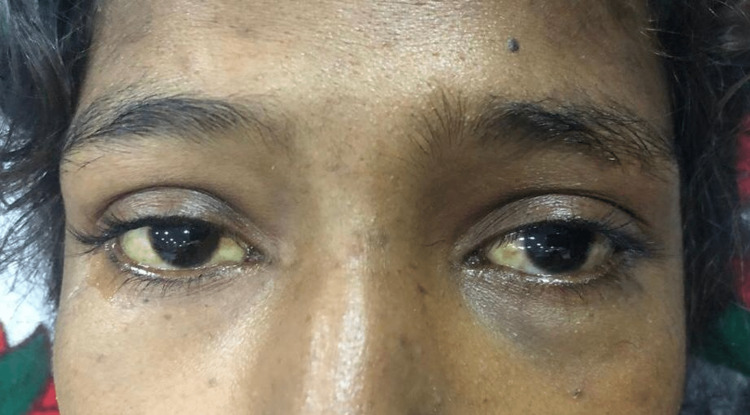
Scleral icterus

**Figure 2:**
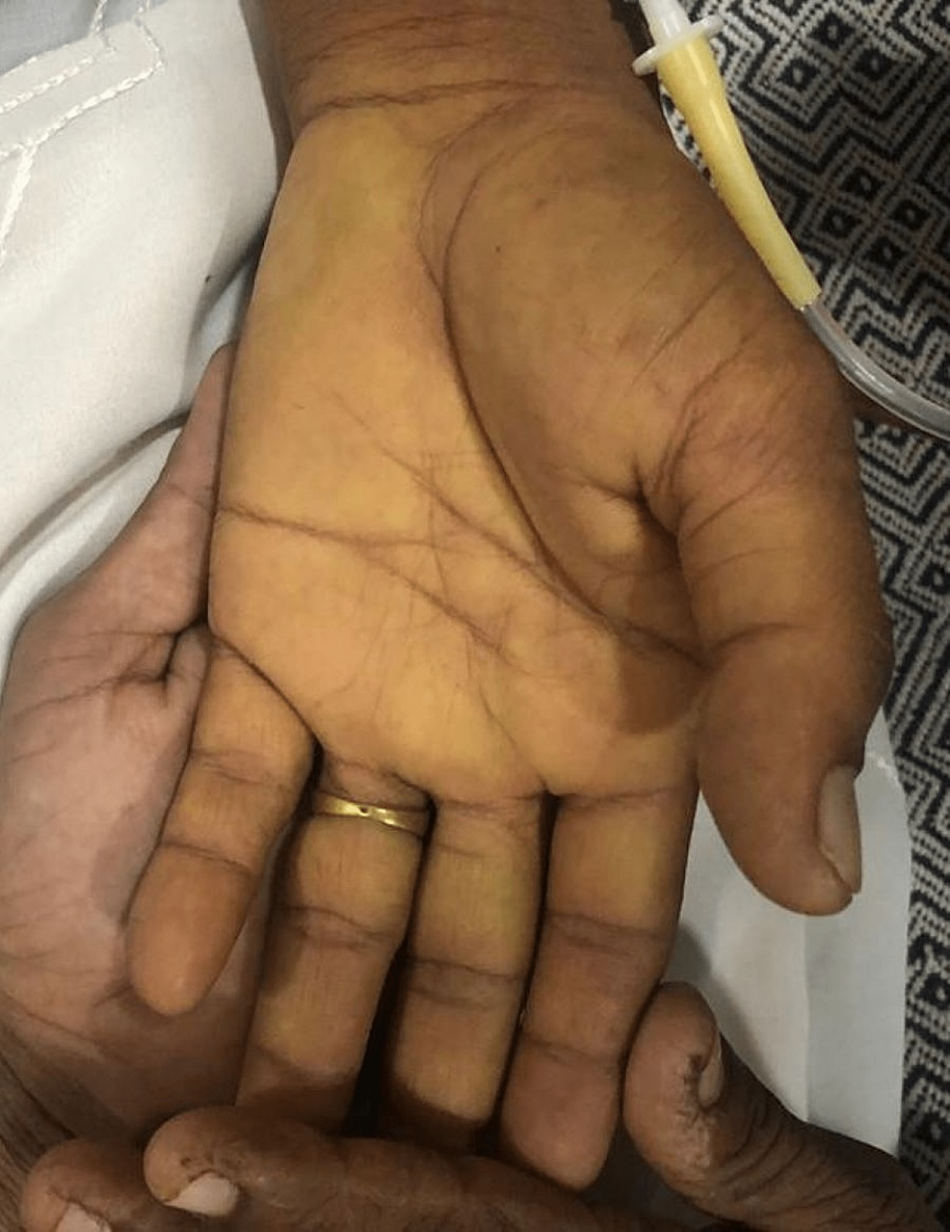
Palmar jaundice

Upon examination, she was lean, alert, and conscious with a Glasgow Coma Scale of 15/15. Her vital status showed a blood pressure of 100/60 mmHg with no postural drop, a heart rate of 87 beats per minute with a regular rhythm, and a respiratory rate of 16 breaths per minute. She was afebrile with a random blood glucose concentration of 108 mg/dl. The patient appeared pale, icteric, and had bilateral pedal oedema of a pitting nature, extending up to ankles. On abdominal examination, the abdomen was sunken with no visible pulsations or scar marks. No tenderness was elicited on superficial and deep palpation. On visceral palpation, no organomegaly was appreciated and there were no signs of free fluid in the abdomen. The rest of her neurological, cardiovascular, and respiratory examinations were normal. No signs of cerebellar and movement disorders were appreciated in the patient. The scleral and palmar icterus are shown in Figure 1 and Figure 2, respectively. 

Her initial laboratory investigations revealed macrocytic anaemia (haemoglobin (Hb) 9.1 g/dl, mean corpuscular volume (MCV) 108 fl), raised corrected reticulocyte count (4.8%), thrombocytopenia (25*10^9), and leukopenia (2.6*10^9 g/dl). The peripheral smear showed schistocytes (small fragmented red blood cells (RBCs)), anisopoikilocytosis with predominant macrocytes, hypochromia, and nucleated red cells. Her liver function tests were significantly deranged showing raised total bilirubin (11.06 mg/dl with a mixed pattern, indirect 6.41 mg/dl, and direct 4.65 mg/dl) and elevated transaminases. Serum lactate dehydrogenase (LDH) was also significantly raised (1048 mg/dl). Her renal function tests showed normal anion gap metabolic acidosis with hypokalemia. Urine studies were significant for urine pH of 5.2, urine anion gap of −2 (urine sodium 82 mmol/L, urine potassium 38 mmol/L, and urine chloride 122 mmol/L, serum osmolality 284 mOsm/kg H2O, urine osmolality 380 mOsm/kg, urine potassium 38 mmol/L, and serum potassium 2.2 mmol/l), and both urine protein and glucose were negative. Her investigations are summarized in Table 1.

**Table 1:** Laboratory results for blood tests Hb= haemoglobin, MCV= mean corpuscular volume, HCT= hematocrit, PLT= platelets, WBC= white blood cells, CRP= C-reactive peptide, ALT= alanine transferase, AST= aspartate transferase, ALP= alkaline phosphatase, GGT= gamma-glutamyl transferase, LDH = lactate dehydrogenase, INR= international normalised ratio, APTT= activated partial thromboplastin time, BUN= blood urea nitrogen, PaO2= partial pressure of oxygen, PaCO2= partial pressure of carbon dioxide, ANA= antinuclear antibodies, AMA= antimitochondrial antibodies, Anti-DS DNA= anti-double-stranded deoxyribonucleic acid antibodies, Anti SS-A/ SS-B= anti-Sjogren's syndrome-related antibodies (A and B), Anti-U1 RNP= anti-ribonucleic protien antibodies, Anti-SCL 70= anti-topoisomerase 1 antibodies, Anti-Sm= anti-Smith antibodies, ASMA= anti-smooth muscle antibodies, Anti-LKM= anti-liver-kidney-muscle antibodies, Anti-SLA= anti-soluble-liver antibodies

Complete blood count	On Admission	10th-day post-admission	Normal values
Hb	9.1g/dl	7.9g/dl	11.9 to 14.8g/dL
MCV	108 fl	110 fl	85 to 98fl
HCT	27.3%	23.7%	35 to 43%
Corrected Reticulocyte count	4.8%	5.1%	0.5-2%
PLT	25*10*9/L	14*10*9/L	150-400 x 10^9/L
WBC	2.6*10*9/L	2.9*10*9/L	3.8 to 10.4*10*9/L
Neutrophil	78%	80%	40% to 60%
Lymphocyte	14.5%	13.6%	20% to 40%
Eosinophil	0.4%	0.5%	1% to 4%
Monocyte	6.2%	4.8%	2% to 8%
Basophil	0.9%	1.1%	0.5% to 1%
CRP	8mg/dl	12mg/dl	Less than 10 mg/L.
Liver Function Tests			
Total bilirubin	11.06mg/dl	23.5mg/dl	0.2 to 1.2 mg/dL
Direct Bilirubin	4.65mg/dl	7.8mg/dl	0.1 to 0.4 mg/dL
Indirect Bilirubin	6.41mg/dl	15.7mg/dl	0.2-0.8 mg/dL
AST	126 U/L	548 U/L	8 to 48 U/L
ALT	195 U/L	763 U/L	7 to 55 U/L
ALP	110 U/L	125U/L	30 to 120 U/L.
GGT	16 U/L	25 U/L	8 to 61 U/L
LDH	1048 mg/dl	1310 mg/dl	122 to 222 U/L
Total protein	5.9 mg/dl	4.8mg/dl	6.3 to 7.9 g/dL
Albumin	2.2mg/dl	1.7mg/dl	3.5 to 5.0 g/dl
Coagulation Profile			
Prothrombin time	14seconds	78seconds	9.4 to 12.5 seconds
INR	1.16	6.5	0.8 to 1.1
APTT	26seconds	47seconds	30-40 seconds
Basic Metabolic Panel			
Sodium	138 mEq/L	132 mEq/L	136-146 mEq/L
Potassium	2.2 mEq/L	2.0 mEq/L	3.5-4.5 mEq/L
Chloride	110 mEq/L	109mEq/L	96-106mEq/L
Creatinine	0.7 mg/dl	0.9 mg/dl	0.6 to 1.1 mg/dL
BUN	8 mg/dl	12mg/dl	6 to 24 mg/dL
Glucose	96mg/dl	66mg/dl	70 to 99mg/dl
Arterial pH	7.23	7.14	7.35-7.45
PaO2	93mmHg	86mmHg	80-100mmHg
PaCO2	36 mmHg	34mmHg	35-45mmHg
Bicarbonate	18 mEq/L	12 mEq/L	22-24mEq/L
Coomb’s Test (direct)	Positive IgG		
Autoimmune profile ANA AMA Anti-dsDNA SS-A SS-B U1-RNP Scl-70 Sm ASMA Anti-SLA Anti-LKM Serum IgG	Negative Negative		
Hepatitis E Serology	Positive IgM		
Serum ceruloplasmin levels	6ug/dl		18 to 35 ug/dL
24-hour Urinary Copper levels	88ug/ 24 hours		20 to 50ug/24 hours
Serum Copper levels	76.6ug/dl		10-15μg/dL

Given her liver function tests and jaundice, the patient was investigated for causes of acute hepatitis. Her hepatitis screening came positive for the Hepatitis E virus (HEV). The patient was worked up for hemolytic anaemia and her direct Coombs antibody test came out strongly positive. Urinalysis was normal. Ultrasound abdomen showed shrunken liver with a coarse echo texture with mild ascites. We evaluated our patient for underlying chronic liver disease (CLD) based on her clinical, laboratory and radiological findings. A complete autoimmune workup including liver autoantibodies was sent with suspicion of autoimmune hepatitis as favoured by secondary amenorrhea and deranged liver function tests in a young female, but the results were negative. Further investigations were done to look for other causes.

As our patient had renal tubular acidosis (RTA) along with CLD, we investigated our patient for Wilson’s disease. A slit lamp examination was performed that revealed Kayser-Fleischer rings bilaterally. Serum ceruloplasmin and 24-hour urinary copper levels were analyzed, which showed a low level (6 mg/dl) of serum ceruloplasmin and raised 24-hour urinary copper excretion (88ug/24 hours). Serum copper levels were done which were elevated (76.6 mg/dl). Magnetic resonance imaging of the brain with contrast was performed which showed normal results with no findings suggestive of copper deposition in the brain. A liver biopsy was planned but due to financial constraints could not have been performed. The patient had the positive findings of Kayser-Fleischer rings on slit lamp examination, with low serum ceruloplasmin levels, raised urinary copper excretion, and high serum copper content, fulfilling four out of five criteria of Leipzig score, enough to confirm the diagnosis of Wilson's disease.

The patient was started on penicillamine and zinc acetate. However, during her illness over ten days, she developed liver failure with hepatic encephalopathy, with a deterioration in her Glasgow Coma Score (3/15), coagulopathy (international normalised ration (INR) 6.5), and worsened liver function tests. Her laboratory results on the tenth day post-admission are shown in Table 1. She was immediately resuscitated with crystalloids. Vitamin K and fresh frozen plasma were administered given her coagulopathy, and the patient was purged for encephalopathy, intravenous antibiotics, and L-ornithine L-aspartate therapy was initiated. The patient was immediately placed on the liver transplant list as the definitive management of her acute liver failure (ALF), but the patient underwent a fatal unfortunate demise while awaiting the transplant.

## Discussion

HEV is a single-stranded ribonucleic acid (RNA) virus that causes inflammation of the liver. The presentation range from asymptomatic to acute hepatitis, fulminant hepatic failure, or cirrhosis in immunosuppressed patients [7]. HEV is distributed worldwide, but most cases are seen in developing countries due to outbreaks of unsafe contaminated water, especially in South Asia [2]. Pakistan still harbours a major burden of HEV. Previous studies have shown that patients who were formerly stable on their underlying CLD can become acutely decompensated with complications, like hepatic encephalopathy and hepatorenal syndrome, and derangements in the coagulation cascade [8]. Of the four genotypes, genotype 1 is the most prevalent in South Asia, especially in Pakistan [9].

Progressive hepatolenticular degeneration or Wilson's disease has numerous manifestations leading to diagnostic difficulties. The European Society of Liver (EASL) in 2012 recommend using the Leipzig score for the diagnosis of Wilson’s disease [10]. The components are Kayser-Fleischer rings, neurological involvement, serum ceruloplasmin levels, Coombs negative hemolytic anaemia, liver and urinary copper and chromosomal mutation. The disease is more prevalent in men with a mean age of presentation of 26 years [11]. Most patients with the disease remain asymptomatic throughout their lives, however, symptomatic patients commonly have hepatic or neuropsychiatric complications [12]. The haematological manifestations of the disease include pancytopenia, isolated cell line dysfunction secondary to hypersplenism, and Coombs negative hemolytic anaemia [6]. The prevalence of hemolysis in patients of Wilson's disease is 17% which is due to the release of unbound copper from the dysfunctional hepatocytes leading to the oxidation of haemoglobin resulting in extravascular hemolysis of red blood cells (RBCs) [13]. However, no cases of Coombs positive hemolytic anaemia in Wilson’s disease have been reported. We report the study of a young female presenting with acute HEV infection and later diagnosed with CLD secondary to Wilson’s disease. The patient developed ACLF according to the European Association of the Study of Liver-Chronic Liver Failure (EASL-CLIF) criteria. Upon investigation, she had Coombs positive hemolytic anaemia. No cause other than Wilson's disease was identified for positive antibodies. However, this finding had no impact on the outcome of our patient.

A case study from England reports a similar case of acute HEV infection in a previously undiagnosed Wilson’s disease in a four-year-old girl who had a resolution of her fulminant hepatic failure with a timely liver transplant [14]. Similarly, a retrospective study carried out in India studied nine participants with ACLF, with HEV being the trigger for acute decompensation. it was observed that six out of nine participants had underlying Wilson’s disease [15]. This makes HEV a common cause of acute decompensation in patients with underlying Wilson’s disease.

## Conclusions

Our case documentation is important as it will guide the attending physicians to diagnose patients with jaundice, worsening liver function tests, and acute liver failure. Laboratory and radiological investigations suggestive of CLD should be investigated for metabolic, autoimmune, and viral causes. Acute precipitating factors and secondary viral infections should be screened. We recommend physicians initiate preventive protocols with vaccination and safe drinking water and sanitation availability. Patient education regarding the decompensation of compensated CLD should be promoted. A recombinant HEV vaccine has been developed and is licenced in certain parts of the world. It is important to conduct further trials and studies to determine the use in at-risk groups with worse outcomes such as women of childbearing age, patients with CLD, immunocompromised, elderly, travellers travelling to an endemic area, and at-risk healthcare professionals. Hemolysis is commonly reported in patients with Wilson’s disease, however, autoimmune hemolysis with Coombs positivity is rare and this is the first case reported to our knowledge. We would recommend further documentation and investigation of autoimmune hemolysis in patients with Wilson’s disease.
